# Association Among the Gut Microbiome, the Serum Metabolomic Profile and RNA m^6^A Methylation in Sepsis-Associated Encephalopathy

**DOI:** 10.3389/fgene.2022.859727

**Published:** 2022-03-30

**Authors:** Hui Wang, Qing Wang, Jingjing Chen, Cunrong Chen

**Affiliations:** Department of Intensive Care Medicine, Union Hospital Affiliated to Fujian Medical University, Fuzhou, China

**Keywords:** sepsis-associated encephalopathy, gut microbiota, serum metabolomic, 16S rDNA, rna m6a

## Abstract

**Objective:** To investigate the relationship among the gut microbiome, serum metabolomic profile and RNA m6A methylation in patients with sepsis-associated encephalopathy (SAE), 16S rDNA technology, metabolomics and gene expression validation were applied.

**Methods:** Serum and feces were collected from patients with and without (SAE group and non-SAE group, respectively, *n* = 20). The expression of serum markers and IL-6 was detected by enzyme-linked immunosorbent assay (ELISA), and blood clinical indicators were detected using a double antibody sandwich immunochemiluminescence method. The expression of RNA m^6^A regulator were checked by Q-RTPCR. The gut microbiome was analyzed by 16S rDNA sequencing and the metabolite profile was revealed by liquid chromatography-mass spectrometry (LC-MS/MS).

**Results:** In the SAE group, the IL-6, ICAM-5 and METTL3 levels were significantly more than those in the non-SAE group, while the FTO levels were significantly decreased in the SAE group. The diversity was decreased in the SAE gut microbiome, as characterized by a profound increase in commensals of the *Acinetobacter*, *Methanobrevibacter*, and *Syner-01 genera*, a decrease in [*Eubacterium*]_*hallii*_group, while depletion of opportunistic organisms of the *Anaerofilum*, *Catenibacterium*, and *Senegalimassilia* genera were observed in both groups. The abundance of *Acinetobacter* was positively correlated with the expression of METTL3. The changes between the intestinal flora and the metabolite profile showed a significant correlation. *Sphingorhabdus* was negatively correlated with 2-ketobutyric acid, 9-decenoic acid, and l-leucine, and positively correlated with Glycyl-Valine *[Eubacterium]_hallii*_group was positively correlated with 2-methoxy-3-methylpyazine, acetaminophen, and synephrine acetonide.

**Conclusion:** The gut microbiota diversity was decreased. The serum metabolites and expression of RNA m6A regulators in PBMC were significantly changed in the SAE group compared to the non-SAE group. The results revealed that serum and fecal biomarkers could be used for SAE screening.

## Introduction

Sepsis-related encephalopathy (SAE) is a severe disease with brain dysfunction, mainly caused by non-central nervous system sepsis (Guo et al., 2021). The clinical manifestation of SAE are a disturbance of consciousness, mild cognitive impairment, delirium, and coma (Tomasi et al., 2017). The fatality rate of SAE is as high as 30–70%, seriously affecting the survival of patients (Kempker and Martin, 2016). At present, electroencephalogram (EEG), transcranial Doppler, and a series of serum markers (including intercellular adhesion molecule-5 [ICAM-5] and soluble protein-100β [S-100β]) have certain value in the early diagnosis, evaluation, prognosis, and other aspects of SAE. However, prospective studies with large samples are lacking, and there is currently no targeted therapy available for the early prevention and symptomatic treatment of SAE (Harding et al., 2016; Falck et al., 2017). SAE is a pathological state, but its pathogenesis is not yet fully understood. The occurrence of SAE is believed to be related to the nonspecific inflammation and noninflammatory response of brain cells. The pathogenic basis of SAE is the change in the metabolic function of brain cells following brain injury (Flierl et al., 2010; Sallam et al., 2016; Khaertynov et al., 2017). Neuroinflammation is the main mechanism which recently are reported related to RNA m^6^A methylation underlying the development of SAE (Ji et al., 2015; Nardelli et al., 2016). Therefore, anti-neuroinflammation could be a key factor in improving this syndrome.

The gut microbiota is involved in the nervous system, apoptosis, immunity, metabolism, blood brain barrier, and other brain functions through the gut-brain axis. Abnormal changes in gut microbes are closely related to brain diseases such as cognitive dysfunction (Erny et al., 2015; Chu et al., 2019). The cholinergic anti-inflammatory pathway is an important pathway through which the intestinal flora affect brain function, and is known as the bacterium-entero-brain axis. Cholinergic anti-inflammatory pathways could regulate inflammatory responses in central nervous system and the peripheral tissues (Ertle et al., 2021; Melo et al., 2021; Wedn et al., 2021). The function of the central choline system is closely related to the higher functions of the brain, such as awakening, learning, memory, sleep, and sensorimotor functions. An excessive inflammatory response is an underlying mechanism of the development of SAE, and studies have shown that the electrical stimulation of cholinergic nerves can reduce the occurrence of SAE by inhibiting the inflammatory response (Wang et al., 2016; Hering and Winklewski, 2017; Hoover et al., 2017).

With the widespread application and continuous development of molecular-based technology, metabolomics analysis has been increasingly applied in various studies, the results of which can lay a theoretical foundation for clarifying the mechanisms of numerous diseases (Hara et al., 2015; Zhu et al., 2019; Bai et al., 2021). From the gut microbiome, tryptophan-derived AHR ligands in the CNS can regulate astrocyte function to inhibit inflammation and neurodegeneration (Rothhammer et al., 2016). Metabolites of intestinal flora, such as neurotransmitter short-chain fatty acids, can participate in neural activation and regulate the synaptic activity of proximal neurons of the intestinal nervous system, which are related to many psychiatric diseases (Sajdel-Sulkowska, 2021; Wang et al., 2021).

In recent years, many studies have shown that RNA m6A epigenetics can participate in the regulation of the occurrence and development of a variety of diseases, and more and more evidence shows that metabolism, intestinal flora and RNA epigenetics build a complex cross regulatory network (Jabs et al., 2020; Luo et al., 2021; Yao et al., 2021; Tang et al., 2022). The main regulatory factors of rnam6a in human body have been gradually revealed, including RNA methyltransferase, demethylase protein and so on. Especially m6A in SAE, there is no research on the relationship between these factors.

In this study, we performed 16S rDNA combined with LC-MS/MS to identify differences in the metabolites in the sera of patients with and without SAE. This study was conducted to study the associations among gut microbiome, metabolites and RNA m6A regulators in SAE, and to give new theoretical support for diagnosis and treatment of SAE.

## Materials and Methods

### Study Subjects

Twenty patients with SAE who were admitted to the Union Hospital affiliated to Fujian Medical University from January 2021 to July 2021 were included in the study group, and 20 patients without encephalopathy and sepsis (non-SAE) who were admitted during the same period were included in the control group.

The patients were classified following an examination of symptoms, signs, blood, biochemistry, and laboratory culture. The inclusion criteria were patients who met the diagnostic criteria for sepsis (patients with confirmed severe sepsis were transferred to the ICU for treatment); patients with SAE confirmed by craniocerebral imaging and EEG; age >18 years old; and patients with no previous history of CNS diseases and complete clinical data. Patients with the following conditions were excluded: combined liver and kidney failure, heart failure, and shock; coagulation mechanism disorders; uremia encephalopathy, drug poisoning, cerebral infarction, cerebral hemorrhage, and cerebral tumor; and severe cognitive impairment.

This study was approved by the Ethical Review Committee of the Union Hospital affiliated to Fujian Medical University, and informed consent was obtained from each study participant.

### Deoxyribonucleic Acid Extraction

Stool sampling cups were used to collect fecal samples from the patients in both groups (*n* = 20 per group). A commercial kit (Tiangen, Beijing, China) was used to extract the microbial genomic DNA from each fecal sample (250–500 mg) (TGuide S96 Soil/fecal genomic DNA).

### Ribonucleic Acid Extraction and Q-RTPCR

The total RNA was extracted according to the instructions of the kit. After extraction, the total RNA was extracted with 20 μL total RNA, including RNA, was obtained by elution with ldepc water (pure water without RNA enzyme). RNA purity and concentration were measured on nanodrop. Reverse transcription kits, primers, probes and real-time quantitative RT qPCR kits were purchased from Applied Biosystems. Take 200 ng of total RNA from each group and add 15 μL Reverse transcription reaction was carried out in the RT reaction system. Real time quantitative RT qPCR was performed by fluorescence quantitative PCR. The total RT qPCR reaction system was 20 μL. Of which 2 × taqman Master Mix 10 μL, cDNA 1 33 μL. TaqMan primer and probe 1 μL and autoclaved deionized water 7 67 μL. Reaction conditions: 95°C for 10 min; 95 °C for 15 s, 60°C for 60 s, 40 cycles. Each reaction is provided with 3 multiple holes, and the difference of CQ value between multiple holes is no more than 1, which is used for data analysis and calculate the average CQ value.

### 16S rDNA Sequencing

The 16S rDNA sequencing experiment was performed using BIOTREE (Shanghai, China). Using the primers 338F and 806R to amplify the V3/V4 region of the 16S rDNA genes. The primer sequences were as follows: F: 5ʹ-ACT​CCT​ACG​GGA​GGC​AGC​A-3ʹ, R: 5ʹ-GGACTACHVGGGTWTCTAAT-3ʹ. The established libraries were inspected first, and the qualified libraries were sequenced with an Illumina NovaSeq 6,000 (Illumina). FastQC (0.11.9), Trimmomatic (version 0.33), UCHIME (version 8.1), USEARCH (version 10.0), QIIME, and R packages (v3.2.0) were used to perform bioinformatic analysis. The data was available in public database PRJCA007583 (https://ngdc.cncb.ac.cn/).

For the metastats analysis, a *t*-test was performed to obtain the *p* and the Q values. Finally, according to the *p* or Q value, the relevant species were screened, and the default value was *p* ≥ 0.05.

The default parameters for LEfSe to detect taxa with rich differences between groups. Only those taxa with a log linear discriminant analysis (LDA) score >4 were ultimately considered.

### Metabolite Extraction

The LC-MS/MS nontarget metabolomics experiment was conducted using BIOTREE (Shanghai, China). Briefly, extract solution (1 volume acetonitrile: 1 volume methanol) was added to 50 μL sample. Then, the sample was rotated for 30 s and sonicated for 10 min, before precipitating the proteins. Finally, the sample was centrifuged to collect the supernatant for the next experiments.

### LC-MS/MS

LC-MS/MS was processed on an UHPLC system (Vanquish, Thermo Fisher Scientific). The UPLC BEH Amide column coupled to the Q Exactive HFX mass spectrometer (Orbitrap MS, Thermo) was used. All steps were common procedure processed by company.

### Data Preprocessing and Annotation

ProteoWizard was used to convert the data into the mzXML format, and the R program package (kernel XCMS) was used for peak recognition, peak alignment, and peak integration. Then, it matches with the BiotreeDB (V2.1) self-built two-level mass spectrometry database for material annotation.

### Enzyme-Linked Immunosorbent Assay

Blood samples were collected from the two groups for ELISA (*n* = 20 for each group). On the first day after the diagnosis of SAE and non-SAE, 5 ml of fasting peripheral venous blood was sampled from the patients in the morning, and low-molecular-weight heparin was inserted into the anticoagulant vacuum vein collection. The blood was obtained by centrifugation and collecting the supernatant. ELISA was used to determine the levels of BDNF, NSE, ICAM-5, and S-100β (i.e., S-100β [F0027-B, F0161-B, F11072-B, F11076-B]; Fankew, Shanghai FANKEL Industrial Co., Ltd., China) in the blood samples. All experiments were repeated three times.

### Detection of Blood Clinical Indicators

The white blood cell (WBC) and neutrophil (NEUT) levels were measured by UniCel^®^ DxH 800 Coulter^®^ (Beckman Coulter, Inc., United States). The procalcitonin (PCT) level was measured by a double antibody sandwich immunochemiluminescence method (VIDAS 30, Shanghai Fengyue Trading Co., Ltd., China), and the interleukin-6 (IL-6) (ab178013, Abcam, United States) level was measured by ELISA. All experiments were repeated three times.

### Statistical Analysis

The measurement data are presented as the mean ± the standard deviation. The enumeration data are described as percentage, and the χ^2^ test was used for inter-group comparison. *p-*values < 0.05 indicated statistically significant differences.

## Result

### Patient Characteristics

**FIGURE 1:**
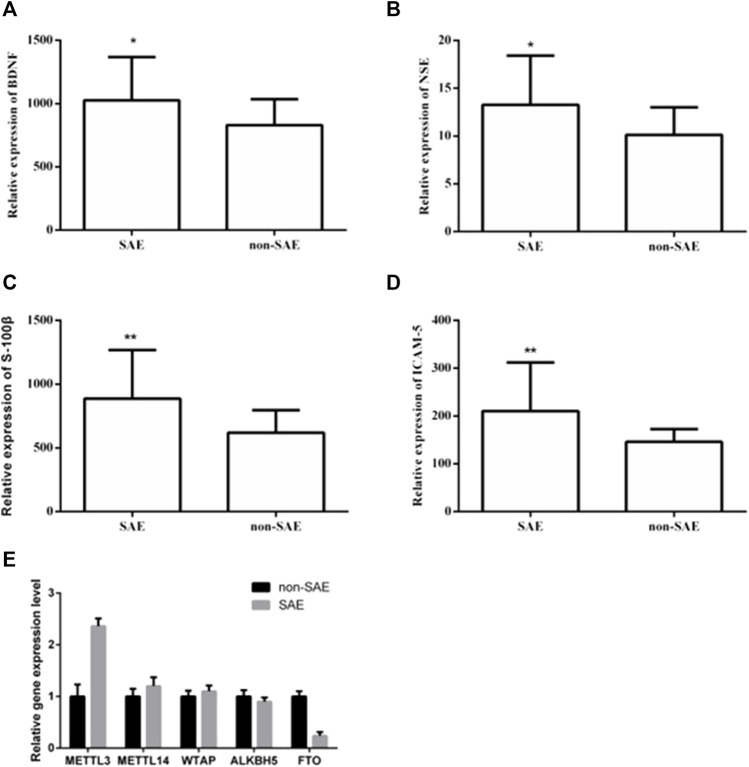
The expression levels of BDNF, NSE, S-100β, ICAM-5, METTL3 and FTO in blood. The expression levels of BDNF **(A)**, NSE **(B)**, S-100β **(C)**, ICAM-5 **(D)**. * and ** indicates *p* < 0.05 and 0.01 respectively.

Twenty patients from each group were selected for clinicopathological analysis. There were no significant differences in sex, age, basic disease (e.g., hypertension and diabetes), or infection site (i.e., respiratory tract, gastrointestinal tract, urinary system, and blood flow) between the two groups (Table 1). The WBC, NEUT, PCT, and IL-6 levels were significantly increased in the SAE group. We used ELISA to further analyze the levels of serum makers in the two groups. In the SAE group, the expression levels of BDNF, NSE, S-100β, and ICAM-5 were significantly higher than those in the non-SAE group (Figures 1A–D). In the SAE group, the expression level of METTL3 was increased while FTO was decreased (Figure 1E). Other m^6^A regulators have no significant difference.

**TABLE 1:** Analysis of general patient data.

	SAE (*n* = 20)	Non-SAE (*n* = 20)	χ2/t/z	*p*
Gender [n/%]			0.107	0.744
Male	13 (65%)	12 (60%)		
Female	7 (35%)	8 (40%)		
Age	62.3 ± 13.55	62.45 ± 12.50	−0.036	0.971
Basic disease [n/%]				
Hypertension	4 (20%)	6 (30%)	0.533	0.465
Diabetes	2 (10%)	3 (15%)	0.000	1.000
Other	17 (85%)	19 (95%)	0.278	0.605
Infection Site [n/%]				
Respiratory Tract	19 (95%)	16 (80%)	0.914	0.342
Gastrointestinal Tract	5 (25%)	5 (25%)	0.000	1.000
Urinary System	1 (5%)	1 (5%)	0.000	1.000
Blood Flow	1 (5%)	1 (5%)	0.000	1.000
Serum Biochemicals				
WBC(×109/L)	15 (9.17)	8 (4.17)	−2.137	0.033*
NEUT (×109/L)	14 (9.17)	7 (3.12)	−2.53	0.011*
PCT (ng/ml)	20 (6.57)	2 (0.8)	−3.194	0.001**
IL-6 (pg/ml)	784 (214,5000)	228 (81,611)	−2.336	0.02*

WBC:white blood cell count; NEUT: neutrophil count; PCT: procalcitonin; IL-6: Interleukin-6. *p < 0.05, **p < 0.01.

### Analysis of the Diversity of Gut Microbiota

**FIGURE 2:**
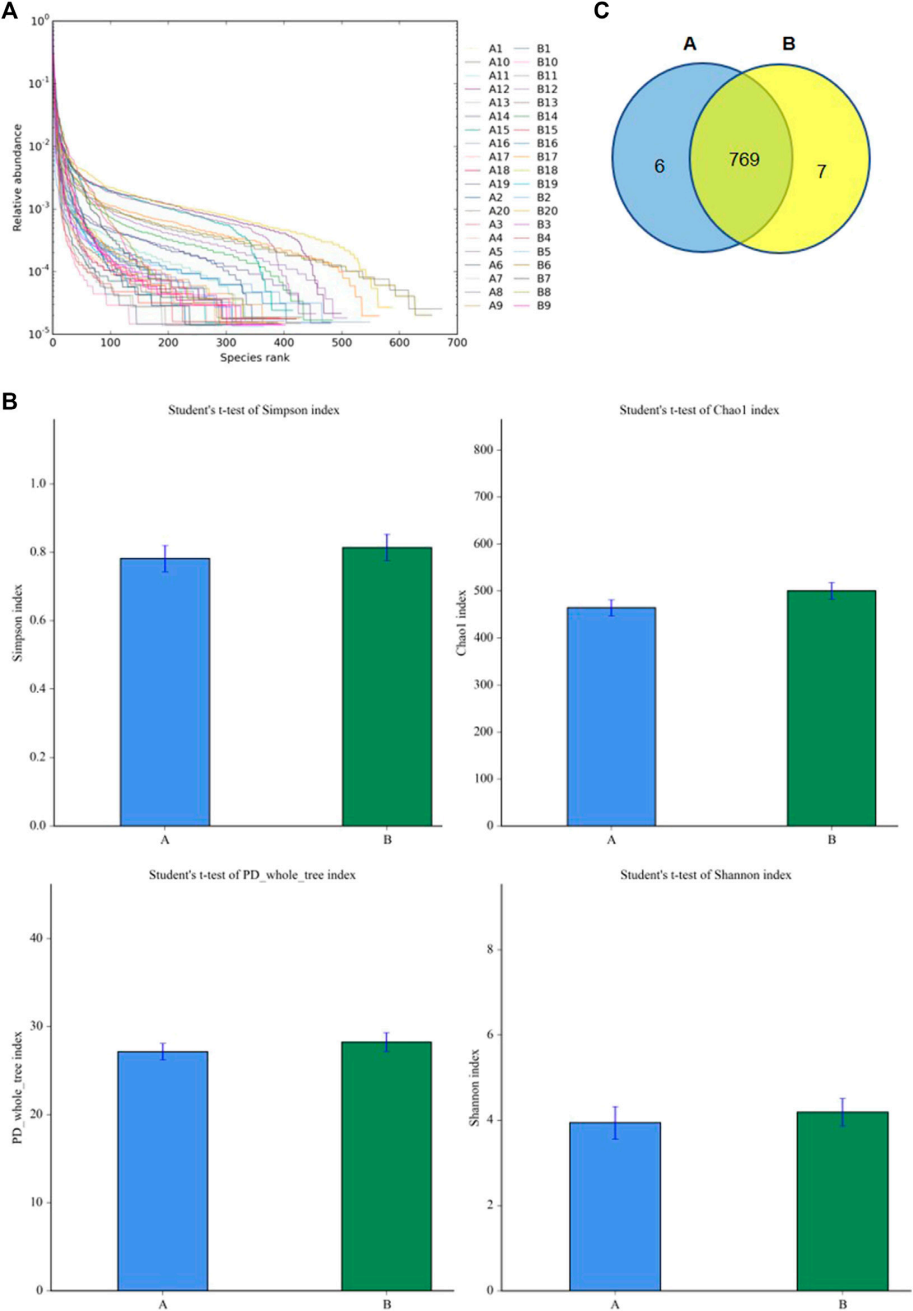
Diversities of fecal microbiome. **(A)** The rank abundance curve. **(B)** The changes of Chao 1 index. **(C)** Venn diagram. A: SAE group; B: nonSAE group.

To understand the diversity of the intestinal microbiota of patients with SAE, the fecal microorganisms in both groups were analyzed by 16S rDNA sequencing. The rank abundance curve showed that all samples contained high species richness and evenness (Figure 2A). The alpha indexes (i.e., Chao1, PD whole, Shannon, and Simpson index) showed that compared to the non-SAE group, the fecal microbial diversity of the SAE group did not significantly decrease (Figure 2B). Analysis of the taxonomic composition on the basis of the OTUs showed 769 common microbial species in the feces of both groups (Figure 2C). Six and seven species-specific microorganisms were found in the SAE and non-SAE groups, respectively (Figure 2C).

### Differences in the Intestinal Microbiota Between the Sepsis-Related Encephalopathy and Non-Sepsis-Related Encephalopathy Groups

**FIGURE 3:**
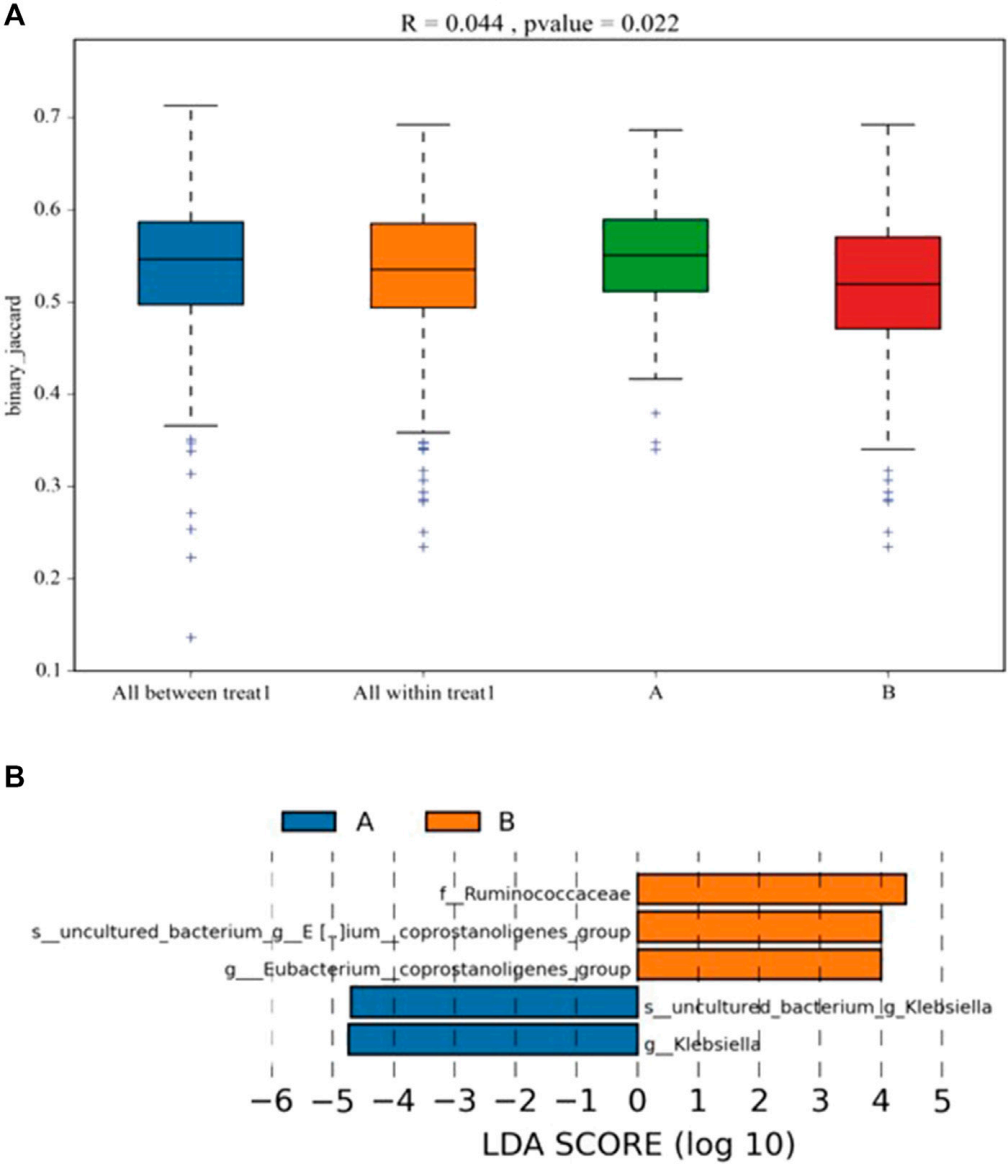
Analysis of the differential microbial in fecal. **(A)** the differentia in two groups (ANOSIM). **(B)** LDA score displays the different microbiota (*p* < 0.05). Orange represents increase, and blue represents decrease. A: SAE group; B: nonSAE group.

ANOSIM was performed to analyze the similarity between multi-dimensional data groups. The R value was 0.044, and the *p* value was 0.022; these values indicated that no significant differences between and within groups, with high inspection reliability (Figure 3A). LEfSe analysis showed five differential biomarkers between the SAE and non-SAE groups (LDA score >4). Compared to the SAE group, the genera *Eubacterium*_*coprostanoligenes*, *Eubacterium*_*coprostanoligenes*_group [*Eubacterium*]_*hallii*_group, and f_Ruminococcaceae were higher in the non-SAE group, while the genera g_*Klebsiella* and s_uncultured_bacterium_g_*Klebsiella* were lower in the non-SAE group (Figure 3B). Metastat analysis revealed 16 different genera between the two groups, showing a remarkable increase in commensals in the *Acinetobacter*, *Methanobrevibacter*, and *Syner-01* but depletion of opportunistic organisms in *Anaerofilum*, *Catenibacterium*, and *Senegalimassilia* in the two groups.

### Serum Metabolomic Profiles of the Sepsis-Related Encephalopathy and Non-Sepsis-Related Encephalopathy Groups

**FIGURE 4:**
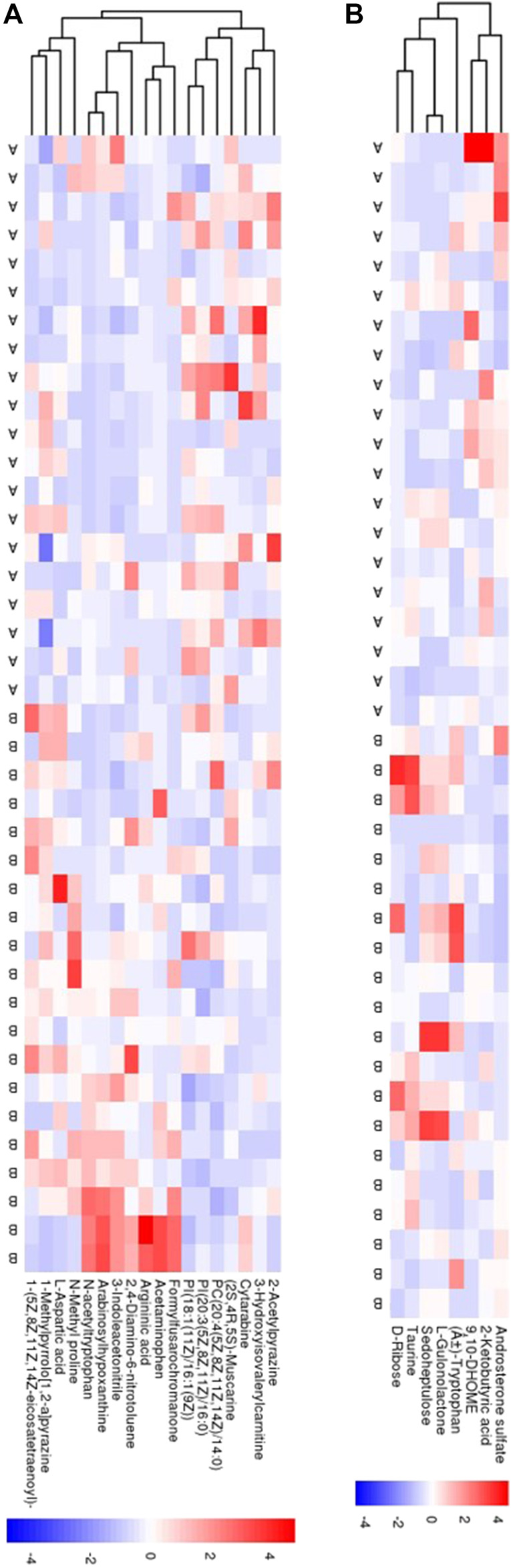
Serum metabolomic analyses. **(A,B)** The PCA score plot and the PLS-DA scores plot in positive ion model and in negative ion mode, respectively. **(C,D)** Heatmaps of 18 metabolites and 8 metabolites in positive ion model and in negative ion model, respectively. Blue circle: QC sample, green circle: nonSAE group, red circle: SAE group. A: SAE group; B: nonSAE group.

The serum metabolic profile was examined using high-throughput LC/MS. The PCA score indicated that clustering of the QC samples in the positive- or negative-ion mode had good stability (Supplemental Figure 1 and 2). The PLS-DA score could separate the SAE group from the non-SAE group according to the difference between the two groups in either the positive- or negative-ion mode (Supplemental Figure 1 and 2). The heatmap showed that in the positive-ion and negative-ion mode, there were 300 (MS2 score >0.8) (Figure 4A) and 158 (MS2 score >0.8) potential biomarkers in the SAE group, respectively (Figure 4B).

### Serum Metabolomic Profiles of the Sepsis-Related Encephalopathy and Non-Sepsis-Related Encephalopathy Groups

**FIGURE 5:**
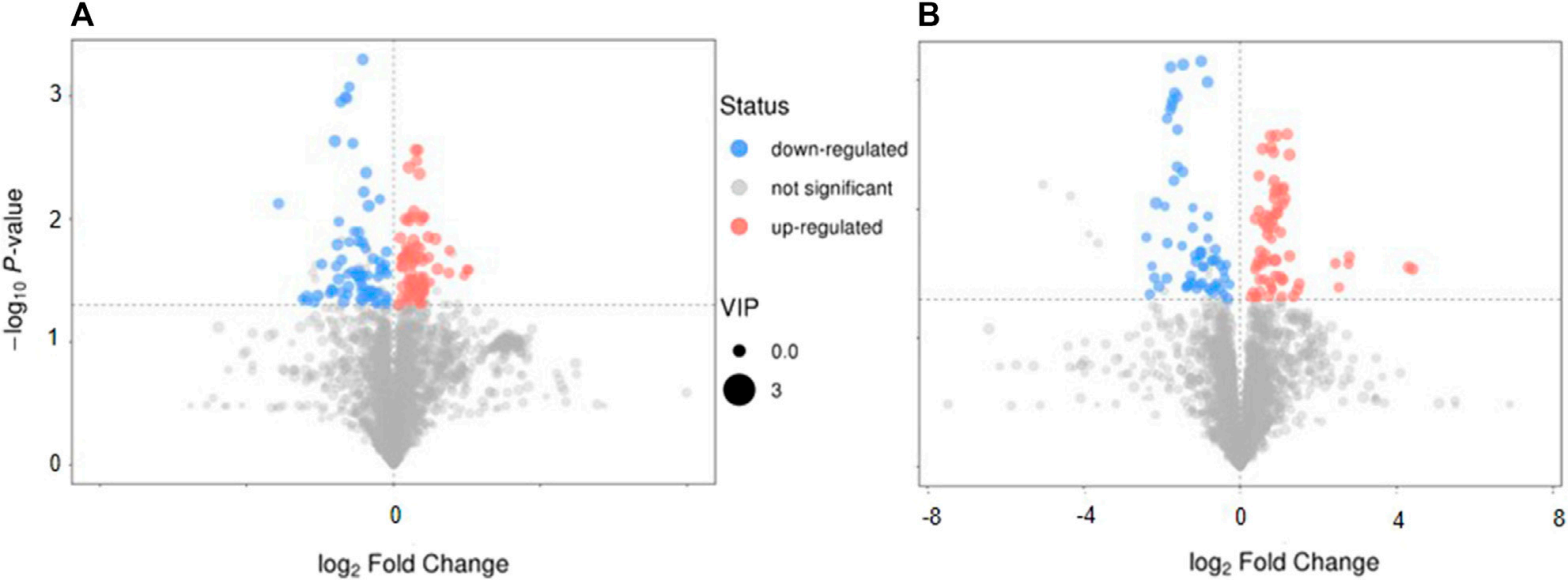
The characteristics and pathway of serum metabolites. **(A,B)** Volcano map in positive-ion model and in negative-ion model, respectively. **(C,D)** The different metabolites pathway related with SAE were confirmed, in positive ion model and in negative ion model, respectively. Red: up-regulation, blue: down-regulation, gray: not significant, *p < 0.05.

The volcano plot showed that 143 of the detected serum metabolites changed significantly in the positive-ion mode (Figure 5A), among which 18 metabolites matched the MS2 name, and 7 and 11 metabolites were downregulated and upregulated, respectively. In the negative-ion mode, 129 of the detected serum metabolites changed significantly (Figure 5B), among which, 8 metabolites matched the MS2 name, and 3 and 5 metabolites were upregulated and downregulated, respectively. In addition, the main metabolic pathways for enriching differential metabolites were analyzed. Thirteen metabolic pathways (Supplemental Figure 3A) (e.g., cyanoamino acid, aspartate, alanine, and pantothenate and glutamate metabolism, and CoA biosynthesis) and six metabolic pathways (Supplemental Figure 3B) (e.g., linoleic acid, taurine, and hypotaurine metabolism and pentose phosphate pathway) were observed.

### Correlation Between Gut Microbiota and Serum Metabolites

**FIGURE 6:**
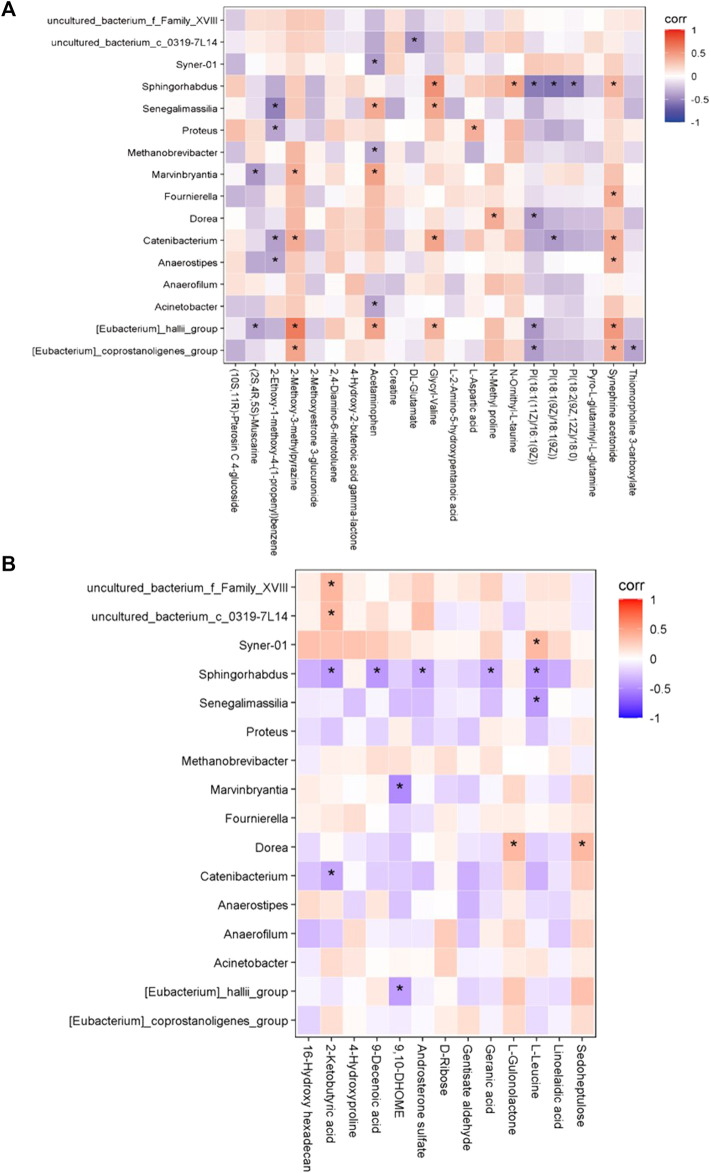
The correlation between gut microbiome and serum metabolomic. **(A)** In positive ion model **(B)** In negative ion model. Red: up-regulation, blue: down-regulation, gray: not significant, *p < 0.05.

We next investigated the possible correlation of changes in the metabolites and the intestinal microbiome spectra. The differences in the intestinal flora and serum metabolites between the two groups were analyzed by Spearman’s correlation coefficient, and were found to have a significant correlation (Figure 6). Furthermore, we found the abundance of *Acinetobacter* was positively correlated with the expression of METTL3, the person correlation factor r was 0.436 with significance (*p* = 0.03).

In the positive-ion mode, the [*Eubacterium*]_*hallii*_group was positively correlated with 2-methoxy-3-methylpyazine, acetaminophen, and synephrine acetonide; *Sphingorhabdus* was positively correlated with glycyl-valine; and the [*Eubacterium*]_*coprostanoligenes*_group was positively correlated with 2-methoxy-3-methylpyazine and synephrine acetonide.

In the negative-ion mode, *Sphingorhabdus* was negatively correlated with 2-ketobutyric acid, 9-decenoic acid, and l-leucine. In addition, uncultured_bacterium_f_Family_XVIII and uncultured_bacterium_c_0319-7L14 were positively correlated with 2-ketobutyric acid. There was a positive correlation between *Dorea* and sedoheptulose, and *Syner-01* and l-leucine.

## Discussion

SAE is a diffuse brain dysfunction, mainly caused by non-central nervous system sepsis. The reasons for this process may include brain inflammation, neurotransmitter dysfunction, and abnormal activation of microglia (Mazeraud et al., 2020).

In our study, the SAE group was found to have a higher expression level of WBC, NEUT, PCT, and IL-6 than the non-SAE group, which was consistent with the findings of previous studies (Jun and Wen Zhenjie, 2020; Zhao et al., 2020; Deng et al., 2021). Studies have shown that the levels of serum NSE, S-100β, and IL-6 were obviously increased in the SAE group; thus, S-100β, serum NSE, and IL-6 levels were significantly correlated with SAE (Tomasi et al., 2017; Guo et al., 2021). A previous study demonstrated that the BDNF levels of patients with SAE were higher than those of patients with sepsis alone (Wen Zhenjie, 2018). In this study, the levels of BDNF, NSE, ICAM-5, and S-100β in the SAE group were obviously increased, consistent with the findings of previous studies. In addition, we found that the expression of mettl3 and FTO changed. Previous studies have shown that RNA m6A is widely involved in the occurrence and development of various diseases. This result also implies its association with metabolism and intestinal flora in diseases. SAE is a pathological state, and its pathogenesis remains ambiguous. At present, it remains necessary to use advanced technology to conduct research to clarify its exact molecular mechanism. This research was performed to better understand the pathogenesis of SAE, and to bring new theoretical support for diagnosis and treatment of SAE. To our knowledge, this is the first study to combine LC–MS/MS metabolomics and 16S rDNA sequencing to analyze the exact molecular mechanism of SAE.

The gut microbiota can regulate the biological processes of the nervous system, apoptosis, immunity, metabolism, the blood brain barrier, and other brain functions through the gut-brain axis. Abnormal changes in these microorganisms are closely related to various brain diseases. With the occurrence of sepsis, the abundance of intestinal flora in the population and rats undergoes specific changes, mainly at the genus level. The proportion of *Alistipes* has risen significantly, contrary to the significant decrease in *Faecalibacterium* (Li et al., 2018). The brain function of rats in the sepsis group decreased with the change in the intestinal flora. Intestinal flora has also been demonstrated to impact SAE via the vagus nerve, with an increase in Firmicutes phylum and a decrease in Proteobacteria phylum observed in the fecal microbiota transplantation groups compared to the lipopolysaccharide group (Liu et al., 2020). Probiotics could protect the sepsis patients from cognitive impairment through reversing the abnormalities in the intestinal flora (Li et al., 2019). Our research revealed that the diversity of the intestinal flora was reduced in the SAE group. In the SAE and non-SAE groups, a substantial increase in commensals in *Acinetobacter*, *Methanobrevibacter*, and *Syner-01* was found, but opportunistic organisms in the *Anaerofilum*, *Catenibacterium*, and *Senegalimassilia* were depleted. The results indicated that the gut microbiota diversity and number were decreased in patients with SAE, which is in line with the results of previous studies.

The host converts intestinal flora metabolites directly or indirectly into nutrients (Mizock, 1990; Jonas et al., 2018). Host cells have various biological functions, and SAE tissue and cell abnormalities can be detected using metabolomics methods, the results of which may contribute to the discovery of new indicators for early diagnosis or therapy of SAE. The concentrations of all aromatic amino acids in cerebrospinal fluid are upregulated in hepatic encephalopathy, whereas in patients with sepsis, only the phenylalanine levels are elevated (Yen et al., 2015). According to the Glasgow Coma Score (GCS), patients with SAE are divided into 15, 12–14, 9–11, and 3–8 groups, with 63 different metabolites observed between the SAE and control groups. The common metabolites in all groups were as follows: for the group with GCS = 15 points, 4-hydroxyphenylacetic acid; GCS = 12–14 points, carbostyril and 3-ethyl-4,7-dimethoxy (35.8%); GCS = 9–11 points, malic acid peak 1; GCS = 3–8 points, oxalic acid. The GCS was also related to the concentration of 4-hydroxyphenylacetic acid (Zhu et al., 2019). In this study, 272 different metabolites and 19 different metabolic pathways were found between the SAE and non-SAE groups. The results showed that the metabolic pathways were abundant in tryptophan metabolism and primary bile acid biosynthesis, which was inconsistent with the results of previous studies.

We found that bacteria and metabolites were correlated in preterm infant feces, and previous studies have shown that bacterial metabolism has an impact on metabolite abundance in humans and mice (Dodd et al., 2017; Stephen et al., 2018). In this study, *Sphingorhabdus* was negatively correlated with 2-ketobutyric acid, 9-decenoic acid, and l-leucine but positively correlated with glycyl-valine. Moreover [*Eubacterium*]_*hallii*_group was positively correlated with 2-methoxy-3-methylpyazine, acetaminophen, and synephrine acetonide. In addition, abundance of [*Eubacterium*]_*hallii*_group was significantly decreased in SAE. This suggest that the correlation may play role in SAE development.

In line with previous studies, our results indicated that the gut microbiota diversity and number were downregulated in the patients with SAE.

## Conclusion

In conclusion, in patients with SAE, the diversity and quantity of intestinal flora were downregulated, and bacteria were increased or depleted, accompanied by changes in the serum metabolic map. Our results uncovered the relationship between intestinal flora and serum metabolites in patients with SAE, which may provide theoretical support for the diagnosis and treatment of SAE.

## Data Availability

The datasets presented in this study can be found in online repositories. The names of the repository/repositories and accession number(s) can be found below: https://ngdc.cncb.ac.cn/, PRJCA007583.
