# Supplementary figures and images for: Association Among the Gut Microbiome, the Serum Metabolomic Profile and RNA m6A Methylation in Sepsis-Associated Encephalopathy
Source: Front Genet. 2022 Mar 30;13:859727. doi: 10.3389/fgene.2022.859727 (PMC9006166; doi:10.3389/fgene.2022.859727)

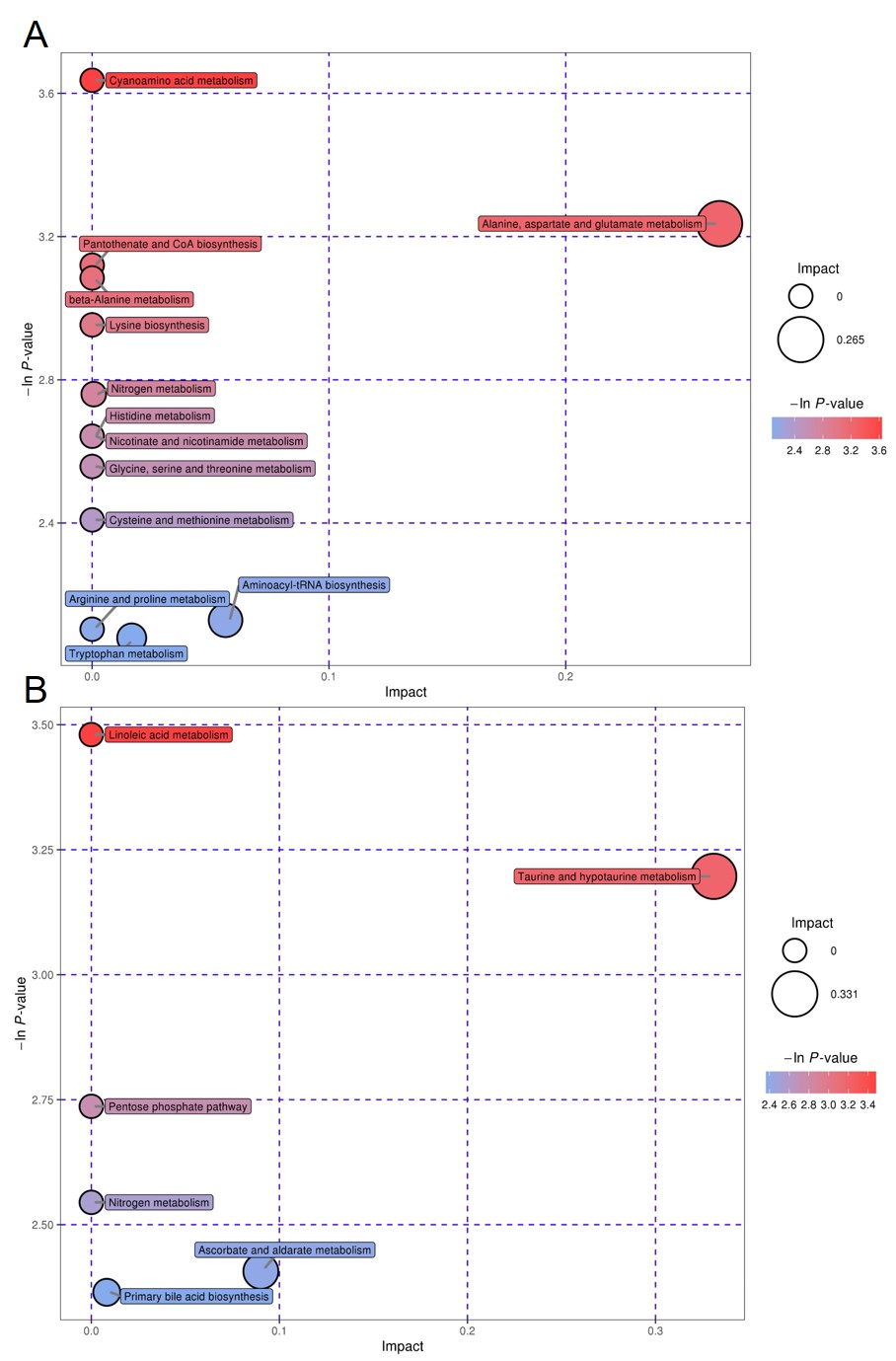

Supplement: Supplementary file 1 [file Image3.jpg]

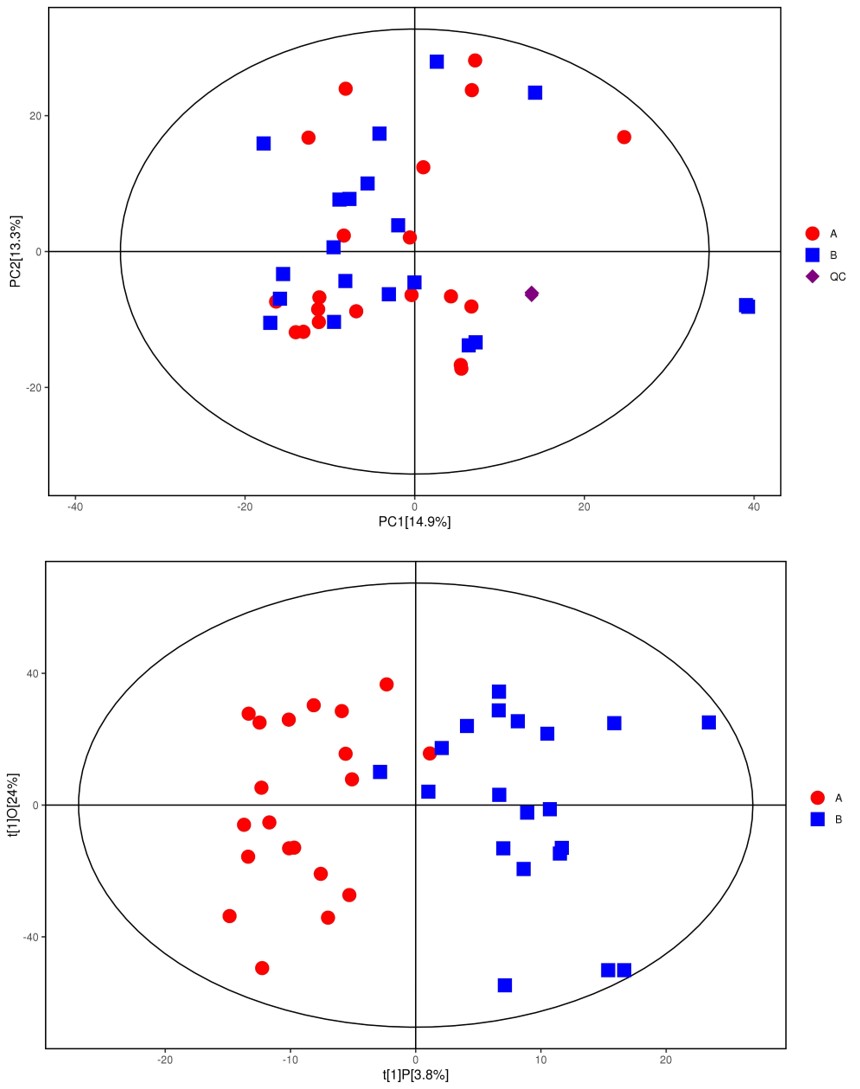

Supplement: Supplementary file 2 [file Image2.jpg]

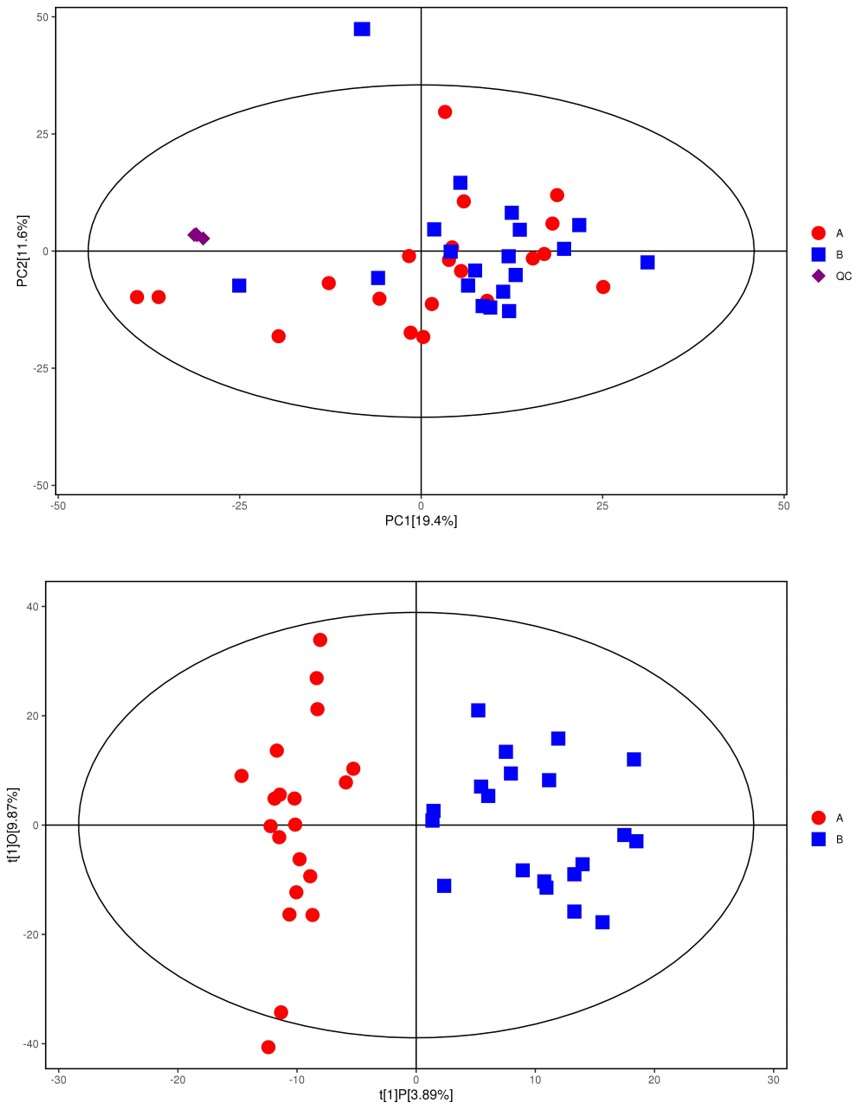

Supplement: Supplementary file 3 [file Image1.jpg]
